# Spleens With Disseminated Lymphoma Exhibit Higher Standardized Uptake Value Maximum, Metabolic Tumor Volumes, and Total Lesion Glycolysis on Fluorine‐18 Fluorodeoxyglucose Positron Emission Tomography/Computed Tomography

**DOI:** 10.1111/vru.70241

**Published:** 2026-09-29

**Authors:** Jesse Johnson, Lynn Griffin, Kathryn Vickery, Elissa Randall

**Affiliations:** ^1^ Veterinary Diagnostic Imaging Department Environmental Health and Radiological Sciences Department Colorado State University Fort Collins Colorado USA; ^2^ Advanced Animal Cancer Imaging LLC Fort Collins Colorado USA; ^3^ Department of Clinical Sciences Flint Animal Cancer Center Colorado State University Fort Collins Colorado USA

**Keywords:** avid, canine, cytology, lymphosarcoma

## Abstract

Standardized uptake value maximum (SUVmax), metabolic tumor volumes (MTV), and total lesion glycolysis (TLG) are metabolic parameters used to quantify tumors detected on fluorine‐18 fluorodeoxyglucose positron emission tomography/computed tomography (18F‐FDG PET/CT). This is a subset project from a larger pilot study where dogs diagnosed with lymphoma via lymph node aspirates were staged and monitored with serial 18F‐FDG PET/CT before, during, and after completion of standard of care chemotherapy. The aim of this prospective study was to evaluate metabolic parameters within the spleen in dogs with naive lymphoma and establish cutoff values for these metabolic parameters to predict lymphoma dissemination to the spleen (*n* = 7 dogs, 17 splenic samples). Splenic contours were performed at each time point to compare splenic cytology to splenic SUVmax, MTV, and TLG. Statistically significant receiver operating characteristic (ROC) curve‐derived cutoff values were found for SUVmax (cutoff > 3.55, *p* ≤ 0.05) and MTV with a threshold of 1.5 SUV (cutoff > 325.7, *p* ≤ 0.05). Additionally, there are significantly higher mean/median splenic SUV, MTV, and TLG among dogs with lymphoma in the spleen compared to those with benign splenic cytology. Therefore, certain cutoff values for SUVmax, MTV, and TLG may be able to predict splenic dissemination. These results may allow for noninvasive staging, monitoring of therapeutic response, and evaluation for recurrence of lymphoma following remission.

## 1 Introduction

Malignant lymphoma is one of the most common cancers in dogs, comprising approximately 7%–24% of all canine neoplasia and 84% of all hematopoietic neoplasia. Lymphoma represents a diverse group of neoplasms that originates from lymphocytes, with multicentric, diffuse large B cell lymphoma often characterized by general lymphadenopathy, being the most common form in dogs [1].

Lymphoma has five stages (see Table 1; [1, 2]) with a worsening prognosis associated with a higher stage and substage. Diagnosis and staging of lymphoma requires orchestration of imaging studies that may include thoracic radiographs and abdominal ultrasound in concert with cytologic assessment of lymph nodes, internal organs, and/or bone marrow, with extirpation and histopathologic evaluation of nodal tissue considered the gold standard for diagnosis and classification [1, 3]. Cytology and/or histopathology are especially important when considering that accurate staging determines prognosis and assists with treatment decisions for both the owner and the clinician. Multidrug chemotherapy protocols, including cyclophosphamide (C), doxorubicin (H, hydroxydaunorubicin), vincristine (O, Oncovin), and prednisone (P) (CHOP), are then initiated on the basis of this information. The fundamental goals of chemotherapy are to induce a complete first remission, to reinduce remission when the patient relapses, and to utilize rescue drugs when the cancer fails to respond to induction or reinduction [1].

**TABLE 1:** World Health Organization clinical staging system for multicentric lymphoma in domestic animals [1, 2].

Stage	Anatomic site
I a/b	Single node or lymphoid tissue in a single organ
II a/b	Many nodes in a regional area
III a/b	Generalized lymph node involvement
IV a/b	Liver and/or spleen involvement
V a/b	Blood and/or bone marrow involvement

*Note*: a: without systemic signs; b: with systemic signs.

In veterinary medicine, serial measurements of lymph nodes using calipers are the clinical standard for assessment of clinical remission in lymphoma cases that have peripheral lymphadenopathy as the principal component of the disease presentation [4]. Clinical remission is often achieved in 80%–95% of dogs, with overall median survival times of 10–12 months following initiation of CHOP chemotherapy. Lymph node evaluation is relatively subjective, dependent on both clinician experience and patient conformation. Palpation of lymph nodes often cannot detect minimal residual disease (MRD), a population that consists of treatment‐resistant neoplastic cells. Most patients will eventually succumb to relapse disease of which the MRD serves as the nidus [1, 5, 6].

Fluorine‐18 fluorodeoxyglucose positron emission tomography/computed tomography (18F‐FDG PET/CT) is a form of noninvasive nuclear imaging that identifies areas of interest that have increased glucose utilization, such as cancer cells. FDG is a glucose analog that is taken up by metabolically active cells, including brain, heart, and salivary glands as well as pathologic sites, including cancer or inflammatory cells [7, 8]. It is attached to a radioisotope (18F) that becomes trapped in these cells, then subsequently decays, producing photons that can be captured for the purposes of imaging and localization using a PET scanner. Because PET/CT fuses anatomy with metabolic physiology, it can be more sensitive than radiographs, ultrasound, or CT for cancer detection [9]. In human oncology, serial imaging with 18F‐FDG PET/CT is the standard of care used to stage, assess response to chemotherapy, and confirm a metabolic remission in lymphoma patients [10, 11, 12, 13]. If metabolic remission is not obtained, then a change in treatment is recommended with the goal to eradicate residual tumor cells and thus improve remission and survival time [10].

Although 18F‐FDG PET/CT is widely used for diagnosing, staging, monitoring, and restaging patients with lymphoma in human medicine, there is limited data regarding splenic lymphomatous involvement and how this may affect patient prognosis [14, 15]. One recent human study demonstrated that 18F‐FDG PET‐CT played a valuable role in differentiating lymphoma and inflammation in patients with high diffuse uptake of 18F‐FDG in the spleen, liver, and bone marrow without increased 18F‐FDG uptake in nonenlarged lymph nodes [14]. Splenic lymphoma without lymphadenopathy is exceptionally rare, and most spleens infiltrated by lymphoma on 18F‐FDG PET/CT will be diffusely avid with normal CT appearance or will have solitary or multiple FDG‐avid splenic lesions [15]. Another recent human study in patients with diffuse large B‐cell lymphoma determined that splenic characteristics, such as focal splenic lesions and pretreatment splenomegaly with baseline 18F‐FDG PET/CT in patients with diffuse large B‐cell lymphoma, may be related to the relapse, with focal lesions considered an independent variable (15). In veterinary medicine, there are only a handful of studies that have evaluated lymphoma with 18F‐FDG PET and/or PET/CT, and none of these studies specifically focused on the spleen [16, 17, 18, 19].

Interpretation of 18F‐FDG PET/CT and assessment of prognosis can utilize metabolic tumor parameters, including maximum standardized uptake value maximum (SUVmax), metabolic tumor volume (MTV), and total lesion glycolysis (TLG) [20, 21, 22]. Standardized uptake value (SUV) is a unitless semiquantitative method of evaluating FDG uptake in a defined region of interest (ROI) and is directly proportional to metabolic activity; the avidity is identified by accounting for concentration of FDG uptake in tissue divided by the amount injected all normalized for patient body weight [8, 23]; MTV represents the volume of a tumor within a defined volume of interest (VOI) with a set SUV cutoff value or 40% of SUVmax (units SUV cm^3^); TLG is the MTV multiplied by the SUV mean within the VOI [23, 24].

The objectives of this study were to evaluate metabolic parameters of the spleen, including SUVmax, MTV, and TLG, in dogs with naïve B cell and T cell lymphoma detected on 18F‐FDG PET/CT and to establish cutoff values for these metabolic parameters to predict lymphoma dissemination to the spleen.

## 2 Materials and Methods

This is a subset study of a larger prospective pilot study evaluating dogs with naïve B cell and T cell lymphoma. This study was approved by the ethics committee of the Colorado State University; the Colorado State University Veterinary Teaching Hospital Clinical Review Board (CRB) approved all study designs, methodology, and client consent forms; all animals for this study were enrolled with informed consent following recruitment from a population of client‐owned dogs that presented to the Colorado State University Veterinary Teaching Hospital.

Dogs were included in the larger study population on the basis of a diagnosis with naïve, multicentric, B cell or T cell lymphoma via a combination of cytology and flow cytometry (FC) [25], planned to undergo treatment with CHOP‐based protocol, deemed healthy enough to undergo multiple prolonged anesthesia events, had a performance status of either 0 or 1 on Day 0, according to the modified VCOG Performance Scheme [26], and signed owner‐informed consent obtained. Dogs were excluded if they had any chemotherapy/CHOP‐based chemotherapy, including prednisone prior to initial imaging, radiation to any site in the body within 3 months of enrollment, comorbidities precluding multiple prolonged anesthetic events, and/or patient is on homeopathic/alternative therapies within 1 day of Day 0 (supplements, such as chondroitin sulfate, vitamins, essential fatty acids, and glucosamine, were permitted).

Prior to all 18F‐FDG PET/CT examinations, animals were fasted for a minimum of 6 hours beforehand, and each patient had a same‐day pre‐scan blood glucose of less than 200 mg/dL. Patients underwent 18F‐FDG PET/CT imaging with a Philips Gemini TF Big Bore 16‐slice PET/CT scanner (Philips North America, Andover, Massachusetts) under general anesthesia with patients positioned in dorsal recumbency. 18F‐FDG (Cardinal Health) at a dose of 0.15 mCi per kilogram was injected intravenously. During the 1‐h uptake period, a whole‐body CT scan (pre‐ and post‐contrast) was obtained for anatomic correlation and attenuation correction, most commonly approximately 15 min prior to the subsequent PET scan. Acquisition parameters included: 120 kV, 100 mAs/slice, 0.75 s rotation time, 0.813 pitch, matrix 768 × 768, FOV 600, and 0.75–1.5 mm (dependent on patient size) × 16 detector width. Data volume acquisitions were performed using 5 mm slice thickness in a standard detail algorithm, and 2 mm transverse images were reconstructed using soft tissue +/− bone algorithms. 350 mg I/mL iohexol (Omnipaque 350; GE Healthcare, Princeton, NJ, USA) was given intravenously at a dose of 2 mL/kg. At 60 min post 18F‐FDG administration, a whole‐body PET scan was obtained (scanned in a caudal to cranial direction, typically using 8 to 9–18.0 cm frames with 1.5–3 min per bed; FOV 576). CT attenuation‐corrected images (CTACs) and non‐attenuation‐corrected (NAC) images of the PET data were reconstructed using the line‐of‐response row‐action maximum likelihood algorithm method.

Baseline 18F‐FDG PET/CT (PET1) was obtained prior to treatment start. Fine needle aspirates (FNA) of lymph nodes, the spleen in most patients, and any concerning accessible extra‐nodal sites, as well as peripheral blood, were obtained for the purpose of tumor burden assessment by FC and polymerase chain reaction for receptor antigen rearrangement (PARR). At the end of Cycle 2 of chemotherapy (1–13 days prior to Cycle 3 at approximately 8 weeks after initiation of therapy), an interim PET (iPET) was obtained, and the same regions were sampled. At the end of therapy, a third PET (EoTPET) was performed (1–4 weeks after completion of therapy at approximately 16–23 weeks after initiation of chemotherapy), and similar sampling parameters were employed. Maximum intensity projections (Figure 1) for each PET/CT examination were evaluated for the presence of abnormalities.

**FIGURE 1:**
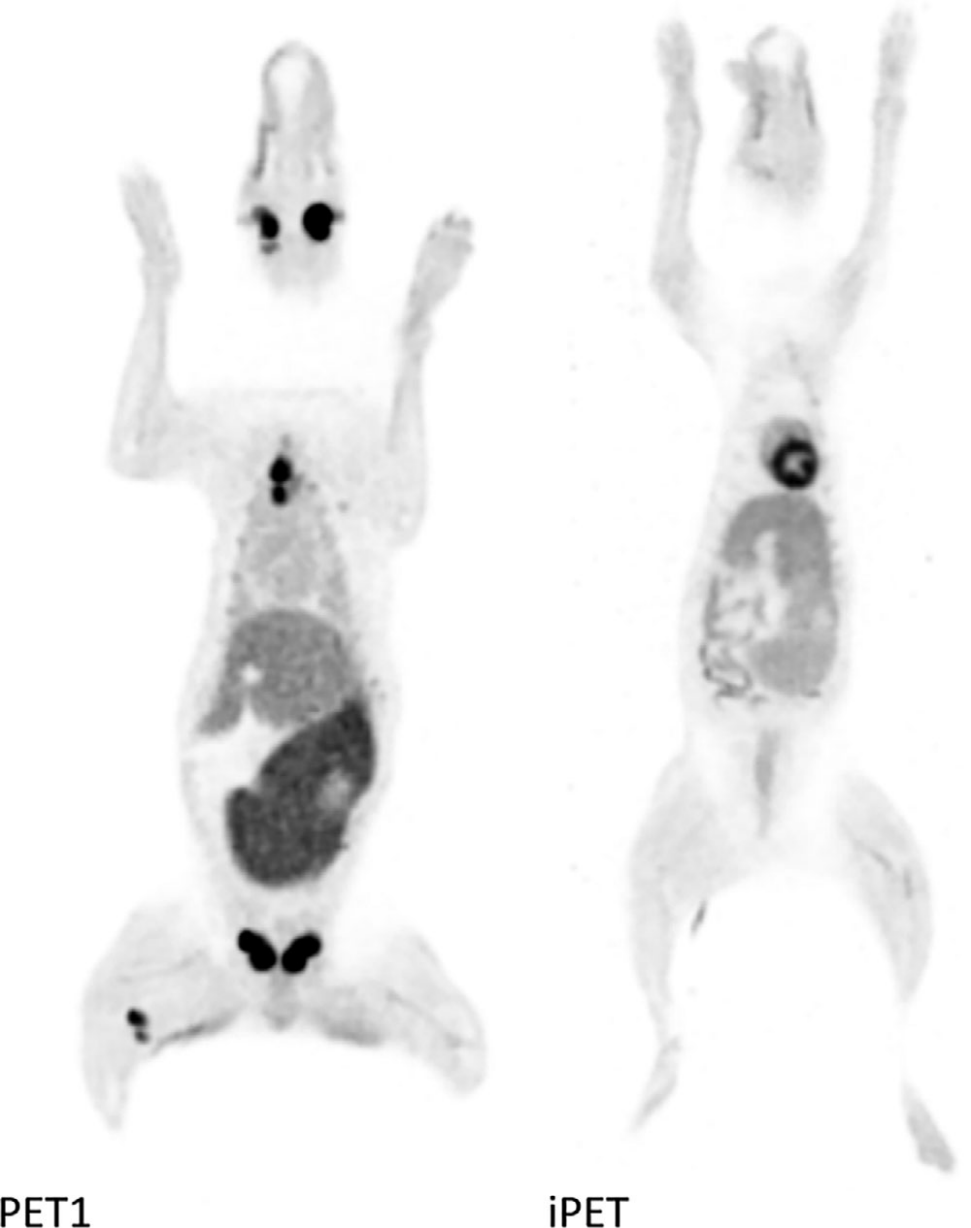
Maximum intensity projections of an 18F‐FDG positron emission tomography/CT dog positive for splenic lymphoma at Baseline (PET1) and negative on interim PET (iPET) following initiation of chemotherapy.

For the purposes of this study, a VOI that was contoured to each patient's spleen, using a threshold of 1.5 SUV, was created and modified in three planes (transverse, sagittal, and dorsal) using Philips IntelliSpace Portal Imaging software [27, 28, 29]. Cytopathologists were blinded to PET results when interpreting samples; the same team of cytopathologists was utilized, although the same cytopathologist did not review all samples. All contours were performed by a second‐year radiology resident (JJ) and confirmed by board‐certified radiologists (ER & LG). Splenic contours and calculated splenic SUVmax, MTV, and TLG were compared to splenic cytology; at the time of image assessment/splenic contouring, the observers were blinded to all staging and splenic cytology results. The following data were also recorded for each patient: age, breed, sex, splenic appearance on contrast CT (separate from PET avidity), and date of each 18 F‐FDG‐PET/CT imaging examination.

## 3 Statistical Analysis

Statistical analysis was performed by a statistician employed by the university. The continuous data on SUVmax, MTV, and TLG was evaluated for normality testing. If data were normal, a *t*‐test was used to compare Lymphoma Yes versus No. If not normal, data were analyzed using Mann–Whitney *U* test. ROC (receiver operating characteristic) curve analysis was performed to determine the cutoff value to classify the Lymphoma positive classification. A *p* value of ≤0.05 was used to determine statistical significance. Fisher's exact test was performed to compare categorical variables if the counts were <5 in at least one of the categories. SAS v9.4 (SAS Institute Inc., Cary, NC) was used for all statistical analyses.

## 4 Results

Seven dogs met the inclusion criteria for this study that was utilizing 18F‐FDG PET/CT for the detection of minimally residual disease in dogs affected with naturally occurring multicentric lymphoma and that had splenic cytology. There were five males and two females with breeds, including a Cane Corso, Cavipoo, Australian cattle dog, and four mixed‐breed dogs with a mean age of 8.5 years old (range: 5–10 years) at the time of first PET/CT.

Of these seven dogs, five had splenic aspirates at all time points coinciding with imaging, two had splenic aspirates at two time points, and one at one time point. There was a total of 17 splenic cytologic samples that were treated as individual variables and correlated with quantitative values obtained from the corresponding PET/CT. Of these 17 splenic samples, 5 were positive (4 at Pet1 and 1 at iPET), and 12 were negative for lymphoma. Four out of five spleens with disseminated lymphoma had a normal appearance on pre‐ and post‐contrast CT examination, prior to fusion with the PET images.

There was a significantly higher splenic SUV, MTV cm^3^ at 1.5 SUV, and TLG at 1.5 SUV cm^3^ among dogs with lymphoma in the spleen (mean and median of 6.58, 4; 605.4, 424.5; and 2064, 811.3, respectively) compared to those with benign splenic cytology (mean and median of 2.59, 2.4; 143.8, 119.5; and 248.5, 200.5, respectively). The *p* value for SUV, MTV, and TLG was 0.0173, 0.0033, and 0.0086, respectively. Figure 2 illustrates examples of varying avidity for spleens positive and negative for lymphoma, and these values are represented in Tables 2, 3, 4.

**FIGURE 2:**
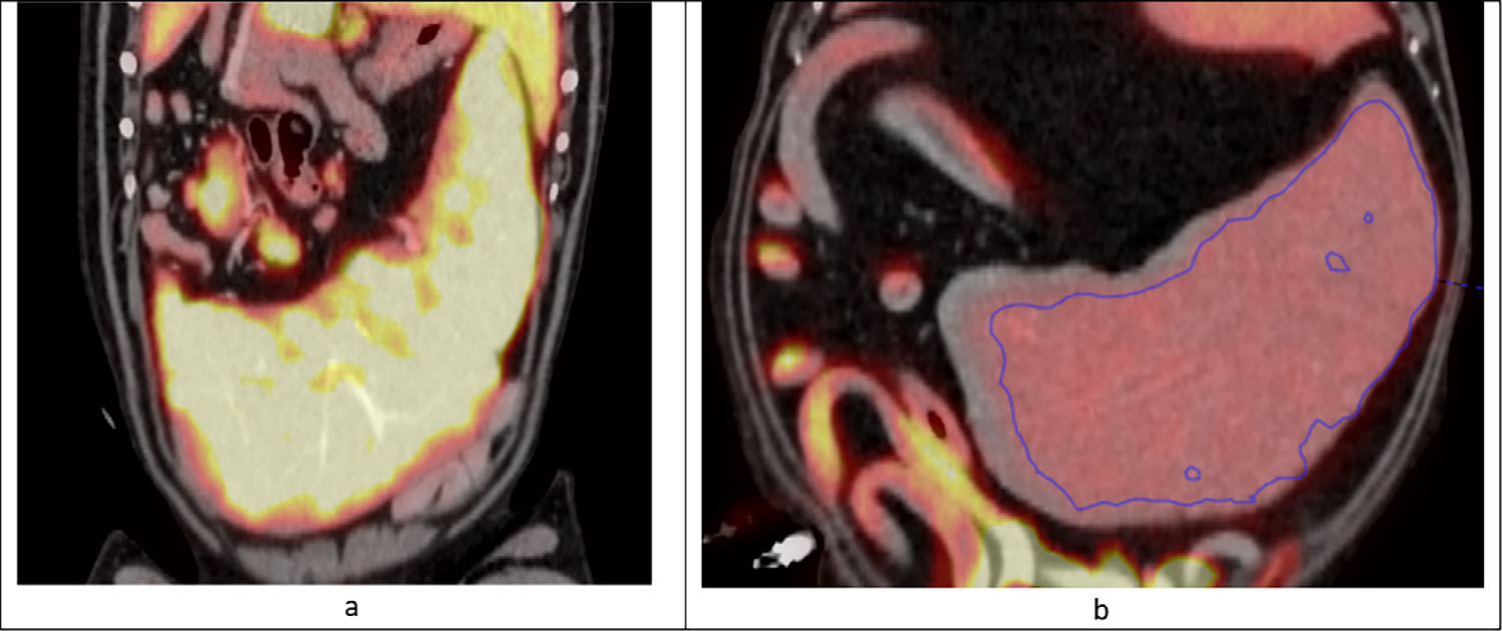
(a) Markedly avid spleen consistent with lymphoma on cytology, SUVmax 18.9. (b) Minimally avid spleen negative for lymphoma on cytology, SUVmax 2.2.

**TABLE 2:** Mean/Median of the acquired standardized uptake value maximum (SUVmax) values for spleens positive and negative for lymphoma.

	*N*	SUV range	Mean	Median
Lymphoma Yes	5	2.7–18.9	6.58	4
Lymphoma No	12	2.0–3.9	2.582	2.4

*Note*: A *p* value of ≤0.05 was used to determine statistical significance. *p* value 0.0173.

**TABLE 3:** Mean/Median of the metabolic tumor volume (MTV) (cm^3^) values for spleens positive and negative for lymphoma.

	*N*	Mean	Median
Lymphoma Yes	5	605.4	424.5
Lymphoma No	12	143.8	119.5

*Note*: A *p* value of ≤0.05 was used to determine statistical significance. *p* value 0.0033.

**TABLE 4:** Mean/Median of the acquired TLG (SUV cm^3^) values for spleens positive and negative for lymphoma.

	*N*	Mean	Median
Lymphoma Yes	5	2064	811.3
Lymphoma No	12	248.5	200.5

*Note*: A *p* value of ≤0.05 was used to determine statistical significance; *p* value 0.0086.

Figure 3 presents the ROC curves that were used to derive the cutoff for SUVmax and MTV with a 95% confidence interval (CI); a cutoff value could not be established for TLG using a Fisher's exact test. At 3.55 cutoff of SUVmax, the likelihood of detecting lymphoma was 16.5 times higher with 95% CI (1.4%–227.1%) and 91.67% specificity (95% CI 64.61%–99.57%) and 60% sensitivity (95% CI 23.07%–92.89%). Similarly, the likelihood of detecting lymphoma was 16.5 times higher when the cutoff MTV value was 325.7 with a 95% CI (1.4%–227.1%) and 91.67% specificity (95% CI 64.61%–99.57%) and 60% sensitivity (95% CI 23.07%–92.89%).

**FIGURE 3:**
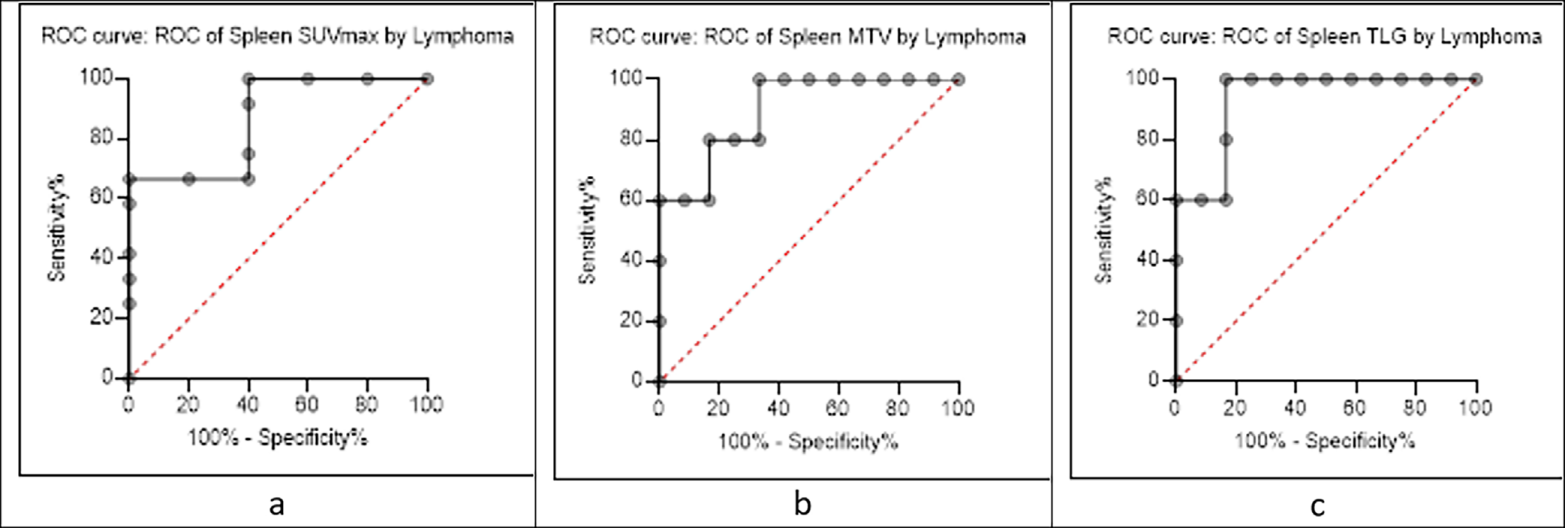
ROC curves to determine the cutoff value to classify the Lymphoma positive classification: (a) ROC of spleen SUVmax by Lymphoma; (b) ROC of spleen MTV by Lymphoma; and (c) ROC of spleen TLG by Lymphoma. MTV, metabolic tumor volumes; ROC, receiver operating characteristic; SUVmax, standardized uptake value maximum; TLG, total lesion glycolysis.

Overall, 100% of the spleens positive for lymphoma demonstrated a diffuse minimal to markedly avid spleen, whereas the spleens negative for lymphoma were characterized by intermittent but smaller, subtotal, contiguous regions, and/or with islands of FDG uptake that were greater than the 1.5 SUV threshold, as demonstrated in Figure 4.

**FIGURE 4:**
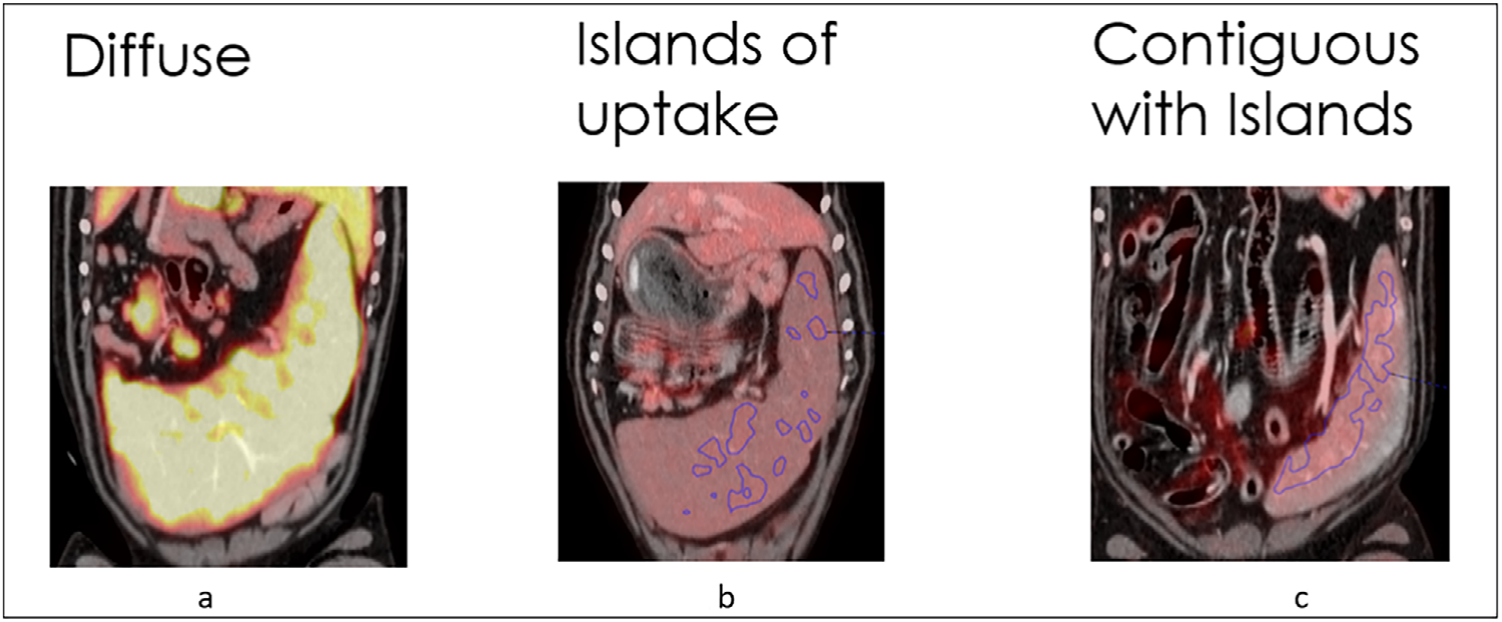
Patterns of distribution for splenic avidity set for a 1.5 SUV threshold: Part (a) demonstrates a diffuse distribution of a lymphoma positive spleen; Part (b) islands of uptake of a lymphoma negative spleen; and Part (c) contiguous with islands of a lymphoma negative spleen.

## 5 Discussion

This study identified significantly higher splenic SUV, MTV at 1.5 SUV (cm^3^), and TLG at 1.5 SUV cm^3^ in spleens with disseminated lymphoma compared to those without for dogs included in this study. An SUVmax of 3.55 and MTV value of 325.7 cm^3^ at 1.5 SUV provided a reasonably sensitive and specific cut off value for prediction of the presence of splenic lymphoma. In veterinary medicine, there have only been limited evaluations of spleens with disseminated lymphoma with PET and/or 18F‐FDG PET/CT and this is the first that has specifically attempted to focus on these quantitative parameters.

Assessment of splenic involvement in lymphoma patients is of critical importance and our study results demonstrate that SUVmax, MTV, and TLG can provide significant insights into the extent of disseminated disease. Often in veterinary medicine, thoracic radiographs and abdominal ultrasound are elected imaging modalities [1]. CT examinations are also frequently used for staging although, in one study, 7/12 livers and spleens with lymphoma appeared normal on CT [30]. Clinicians may under stage lymphoma patients if splenic cytology is not performed based on normal splenic CT or ultrasound imaging findings, such as splenic size, shape, and/or margination. In fact, in our study, four out of five spleens positive for lymphoma on cytology were subjectively normal in appearance on pre‐ and post‐contrast CT but exhibited significantly increased avidity on the 18F‐FDG PET/CT, quantified objectively through measurement of SUVmax, MTV, and TLG. This underscores the utility of 18F‐FDG PET/CT to image metabolic physiology in otherwise anatomically unremarkable structures.

Calculation of MTV and subsequently TLG requires that a cutoff value be established. In many reported cases this value is set at 2.5 SUV or 40% of SUVmax, meaning that all the voxels within the VOI that measure greater than this threshold are included in the volume calculation [23, 27, 31, 32]. Despite the wide use of 2.5 SUV as a cutoff, a multitude of studies have evaluated other cutoff values for various tissues and SUV measurements are affected by many parameters, which may limit the validity of defaulting to 2.5 SUV [27, 29, 33]. In this particular study, a threshold value of 1.5 SUV was utilized. During initial evaluation of the splenic contours for SUV, MTV, and TLG, a variety of thresholds were initially employed, including 40% SUVmax, however, using values higher than a 1.5 threshold resulted in many spleens having no discernible MTV. This decision was supported by previous veterinary studies that have identified splenic SUV in both normal and spleens with disseminated disease to be less than 2.5, including a SUV max in normal canine spleens of 0.94 [34], one identifying a SUVmean of 1.2 in dogs with various types of neoplasia not directly involving the spleen [35] and another that found one spleen with mastocytosis had an SUV max of 1.74, whereas another with a splenic mass had an SUVmax of 10.53 in dogs with mast cell disease [21].

There are a multitude of studies in human medicine that have focused on 18F‐FDG PET/CT with lymphoma staging. One 2009 study demonstrated that the sensitivity and specificity of PET/CT for initial staging of splenic involvement can be 100% and 95%, respectively, with the combined PET/CT demonstrating higher sensitivity and specificity than separate PET or CT scans alone [36]. Another recent human study demonstrated that PET/CT played a valuable role in differentiating splenic lymphoma from inflammation in patients that did not have peripheral lymphadenopathy [14]. Despite 18F‐FDG PET/CT being a common imaging modality for diagnosis and treatment of diffuse large B‐cell lymphoma in human medicine, the evaluation of metabolic parameters such as MTV and TLG as they pertain to patient outcomes remain inadequately explored [24].

Some recent human studies have attempted to explore these metabolic parameters. For example, a 2015 human study evaluating 18F—FDG PET/CT in cases of mantle‐cell lymphoma found that certain cutoff values for SUVmax and TLG at time of diagnosis as well as changes in SUV following treatment can be predictive parameters for patient outcome; their study identified that an SUVmax (</>11.4) at diagnosis and ΔSUV (</>29.65%) from the area in the body with the highest uptake were found to be predictive parameters for patient outcome [37]. A recent study from 2024 evaluating adults with large B‐cell lymphoma for splenic dissemination identified a SUVmax of 3.48 for 9/11 spleens with lymphoma [38]. This study also determined that taking positive spleen TLG and SUVmax is recommended at time of diagnosis and that diagnostic accuracy improves with the assessment of the change in spleen TLG and SUVmax at end of induction chemotherapy. Another 2024 study in pediatric Hodgkin‐lymphoma patients demonstrated that both MTV and TLG from baseline and interim FDG PET scans showed strong prognostic utility for treatment response and event‐free survival [39]. Another recent 2024 study of diffuse large B‐cell lymphoma in adults found TLG of the liver to be a robust prognostic indicator for patients; in this study, patients with low baseline TLG exhibited significantly better outcomes compared to those with higher baseline TLG measurements [24]. This study also identified that patients with initially high TLG were stratified even before treatment with those having the highest initial TLG then having a smaller change in TLG following treatment, ultimately had a worse prognosis. All these studies showed that the initial values of the selected metabolic parameter—SUVmax, MTV, and/or TLG—may improve diagnostic accuracy and/or could predict treatment outcome, with the change of the parameters between studies capable of gauging response to therapy.

As previously discussed, there are limited veterinary studies exploring 18F‐FDG PET and/or PET/CT in lymphoma patients with none that have specifically focused on the spleen [16, 17, 18, 19]. Additionally, to the authors’ knowledge, there have been no veterinary studies that have specifically evaluated SUVmax, MTV, and TLG and their utilization for prognosis in canine lymphoma patients. Recent veterinary studies evaluating dogs with appendicular osteosarcoma found that SUVmax, MTV, and TLG may be prognostic for survival in this patient population [20, 21, 23]. Although our study goals did not directly compare the metabolic parameters to prognosis and treatment outcomes as discussed in the reviewed recent human and veterinary studies, our study found that larger SUVmax, MTV, and TLG values of the spleen were associated with more advanced lymphoma disease stages.

This study indicates that 18F‐FDG PET/CT may be useful as noninvasive method to stage and restage our canine patients with disseminated lymphoma. The established cut off values for SUVmax, MTV, and TLG may assist in identifying those patients that are Stage 4 (liver and/or spleen involvement) or higher. This may guide therapeutic choices and/or recommendations for additional cytologic sampling in a more accurate way than making decisions based on serial caliper measurements of peripheral lymph nodes. This has been demonstrated in human lymphoma patients that have nonenlarged peripheral lymph nodes but disseminated splenic lymphoma [14]. The 18F‐FDG PET/CT metabolic parameter levels may eventually help to guide decisions surrounding treatment without the requirement of cytology. A recent splenic cytology study found an accuracy of 73.08% (95% CI 61.84%–82.50%), sensitivity of 64.29% (95% CI 50.36%–76.64%), and specificity of 95.45% (95% CI 77.16%–99.88%) in identifying splenic pathology [40]. These cytology results demonstrate similar sensitivity and specificity to our study's 18F‐FDG PET/CT metabolic parameter cutoffs with a 3.55 SUVmax cutoff and a 325.7 MTV cutoff, the likelihood of detecting lymphoma was 16.5 times higher with 95% confidence interval and 91.67% specificity and 60% sensitivity.

Some limitations of this study should be acknowledged. This is a single‐center study and SUVmax, MTV, and TLG are variables dependent on image acquisition timing, study protocol, and software programs which can make exact replication challenging. Additionally, there is no gold standard for determining the threshold for the MTV which can lead to variation in MTV and TLG even in the same patient [24]. Further, the small number of patients and even smaller number of spleens positive for lymphoma in this study may impact the statistical power. No histopathologic biopsy results were available and instead, fine‐needle aspirates for cytology were used. Although cytology confirmed lymphoma in five spleens across the three time point scans, it is possible that there were false negatives which could skew our interpretation of the metabolic parameters and the conclusions drawn. In one patient, a computer error barred evaluation of the third PET/CT precluding acquisition of the SUVmax, MTV, and TLG after the initial real‐time read of the study; this limited our ability to evaluate the trend of the metabolic parameters over the course of treatment for this patient. Lastly, all splenic contours in three planes were created manually to remove any additional abdominal or extra‐abdominal structures that had an SUV above the 1.5 threshold. This process was time‐consuming and could be prone to both repeated observer and interobserver variability. Lastly, there is currently limited availability for PET/CT in veterinary medicine which effectively limits its widespread use as a staging method for canine patients.

Our study findings set an exciting stage for future research opportunities utilizing 18F‐FDG PET/CT to diagnose, prognosticate, and both guide and amend therapeutic decisions in canine patients with multicentric B and T cell lymphoma. Additional work on the evaluation of metabolic parameters as they relate to prognosis should be explored. This work can be expanded to include other structures, such as lymph nodes, liver, and/or other avid sites as guided by the PET/CT examination. Specifically evaluating ∆SUVmax, ∆MTV, and ∆TLG in a parallel manner to recent human study designs may strengthen our understanding and provide a basis for better predicting how individual veterinary patients will respond to CHOP and/or rescue protocols as well as potentially predict time to relapse. This study was not focused on prognosis for the patients included. Given this study is a subset of a larger pilot study, some information may be gleaned regarding prognosis and treatment outcomes when splenic cytology along with other tissues are evaluated over the three 18F‐FDG PET/CT scans (prior to initiation, treatment interim, and the end of therapy). Additional evaluation of the spleen at certain time points correlated with additional tissues samples, such as lymph nodes, may further expand our knowledge of the prognostic utility of SUVmax, MTV, and TLG at various time points.

## 6 Conclusion

It was determined that dogs with lymphoma in the spleen (Stage 4) exhibited significantly higher values for SUVmax, MTV at 1.5 SUV, and TLG at 1.5 SUV cm^3^. Certain cutoff values, including 3.55 for SUVmax and 325.7 for MTV, may be able to predict splenic dissemination of lymphoma with a high specificity and moderate sensitivity. These results may allow for noninvasive staging, monitoring of therapeutic response, and evaluation for recurrence of lymphoma following remission. Future prospective studies are necessary to validate and expand on these findings.

## Author Contributions

**Elissa Randall**: conceptualization, investigation, writing – original draft, writing – review and editing visualization, validation, methodology, software, formal analysis, project administration, resources, supervision, credit, data curation. **Jesse Johnson**: conceptualization, investigation, methodology, software, formal analysis, project administration, data curation, validation, visualization, writing – review and editing, writing – original draft, resources. **Lynn Griffin**: conceptualization, investigation, funding acquisition, writing – original draft, writing – review and editing, visualization, validation, methodology, software, formal analysis, project administration, resources, supervision, data curation. **Kathryn Vickery**: conceptualization, investigation, funding acquisition, writing – original draft, writing – review and visualization, validation, methodology, software, data curation, supervision, resources, project administration, formal analysis .

## Disclosure

The abstract was presented at the Fall 2024 ACVR Scientific Conference and at the 2025 CSU Research Day 1/25/2025.

## Conflicts of Interest

The authors declare no conflicts of interest.

## Data Availability

The data that supports the findings of this study are available from the corresponding author upon reasonable request.
